# Interferon-γ Induces Expression of MHC Class II on Intestinal Epithelial Cells and Protects Mice from Colitis

**DOI:** 10.1371/journal.pone.0086844

**Published:** 2014-01-28

**Authors:** Christoph Thelemann, Remzi Onur Eren, Manuel Coutaz, Jennifer Brasseit, Hanifa Bouzourene, Muriel Rosa, Anais Duval, Christine Lavanchy, Vanessa Mack, Christoph Mueller, Walter Reith, Hans Acha-Orbea

**Affiliations:** 1 Department of Biochemistry, University of Lausanne, Epalinges, Switzerland; 2 Institute of Pathology, University of Bern, Bern, Switzerland; 3 Unilabs Pathologie Suisse Romande, Epalinges, Switzerland; 4 Department of Pathology and Immunology, University of Geneva, Geneva, Switzerland; Université Libre de Bruxelles, Belgium

## Abstract

Immune responses against intestinal microbiota contribute to the pathogenesis of inflammatory bowel diseases (IBD) and involve CD4^+^ T cells, which are activated by major histocompatibility complex class II (MHCII) molecules on antigen-presenting cells (APCs). However, it is largely unexplored how inflammation-induced MHCII expression by intestinal epithelial cells (IEC) affects CD4^+^ T cell-mediated immunity or tolerance induction *in vivo*. Here, we investigated how epithelial MHCII expression is induced and how a deficiency in inducible epithelial MHCII expression alters susceptibility to colitis and the outcome of colon-specific immune responses. Colitis was induced in mice that lacked inducible expression of MHCII molecules on all nonhematopoietic cells, or specifically on IECs, by continuous infection with *Helicobacter hepaticus* and administration of interleukin (IL)-10 receptor-blocking antibodies (anti-IL10R mAb). To assess the role of interferon (IFN)-γ in inducing epithelial MHCII expression, the T cell adoptive transfer model of colitis was used. Abrogation of MHCII expression by nonhematopoietic cells or IECs induces colitis associated with increased colonic frequencies of innate immune cells and expression of proinflammatory cytokines. CD4^+^ T-helper type (Th)1 cells - but not group 3 innate lymphoid cells (ILCs) or Th17 cells - are elevated, resulting in an unfavourably altered ratio between CD4^+^ T cells and forkhead box P3 (FoxP3)^+^ regulatory T (Treg) cells. IFN-γ produced mainly by CD4^+^ T cells is required to upregulate MHCII expression by IECs. These results suggest that, in addition to its proinflammatory roles, IFN-γ exerts a critical anti-inflammatory function in the intestine which protects against colitis by inducing MHCII expression on IECs. This may explain the failure of anti-IFN-γ treatment to induce remission in IBD patients, despite the association of elevated IFN-γ and IBD.

## Introduction

IBDs are chronic and recurring inflammatory disorders affecting the human gastrointestinal tract. There are two major clinical forms of IBD, Crohn’s disease and ulcerative colitis. Progression of Crohn’s disease is mainly driven by CD4^+^ Th1 and Th17 cells, and IFN-γ is a signature cytokine of the disease [1], [2].

MHCII-mediated antigen presentation is fundamental for driving CD4^+^ T cell orchestrated immune responses. MHCII is primarily expressed on professional APCs, which induce both effector T cell activation and FoxP3^+^ Treg cell-mediated tolerance [3]. However, under inflammatory conditions, MHCII is typically also induced on nonhematopoietic cells [4]. IECs are able to process and present gut luminal antigens as they express the MHCII antigen-presentation machinery and antigens in the context of MHCII molecules [5], [6], [7], [8], [9], [10]. However, as it remains debated whether IECs are able to provide sufficient costimulation for immunogenic T cell activation [11], [12], [13], it is controversial whether their function as nonprofessional APCs promotes CD4^+^ T cell-dependent tolerance or boosts immune responses *in situ*. Previous observations obtained mainly from *in vitro* or *ex vivo* studies might not reflect the more complex situation *in vivo* [14], [15], [16].

Cell-type-specific expression of the MHCII antigen-presentation machinery is directed by the class II transactivator (CIITA). CIITA expression is tightly regulated by the differential usage of three independent promoters, pI, pIII and pIV [4]. pI is active in myeloid cells. pIII activity is mainly restricted to lymphoid cells. Importantly for this study, pIV−/− mice display a selective abrogation of inducible MHCII expression in nonhematopoietic cells, including IECs. These mice lack positive selection of CD4^+^ T cells due to the absence of MHCII on cortical thymic epithelial cells (cTECs) [17]. However, CD4^+^ T cell development is restored by introducing a CIITA transgene (Tg) driven by the keratin-14 (K14) promoter: The resulting pIV−/− K14 CIITA Tg mice harbour normal repertoires of CD4^+^ T cells and display normal levels of MHCII on professional APCs, which depend on pI and pIII, but lack inducible MHCII expression on nonhematopoietic cells [4], [17], [18].

To investigate the *in vivo* role of nonhematopoietic MHCII expression on the outcome of gut-specific immune responses and pathology, we administered interleukin (IL)-10 receptor-blocking antibodies (anti-IL10R mAb) to pIV−/− K14 CIITA Tg and control mice infected chronically with *H. hepaticus*. In comparison to the control mice, the absence of epithelial MHCII expression led to colitis characterised by enhanced colonic infiltration of innate effector cells and elevated expression of proinflammatory chemokines and cytokines. This resulted in increased infiltration of inflammatory CD4^+^ Th1 cells and an increased ratio between CD4^+^ T cells and FoxP3^+^ Treg cells. A deficiency in IFN-γ, or its neutralization, resulted in the absence or reduced levels of epithelial MHCII expression, respectively, suggesting that mainly T cell-derived IFN-γ is required to induce epithelial MHCII expression. These findings reveal a critical role of IFN-γ-induced epithelial MHCII expression in contributing to intestinal homeostasis by exerting an anti-inflammatory effect *in vivo*, which is consistent with the failure to attenuate IBD with anti-IFN-γ treatment.

## Materials and Methods

### Animals

Mice were on a C57BL/6 background and were used between 6–10 weeks of age. pIV−/− K14 CIITA Tg mice were described previously [18]. C57BL/6 mice were obtained from Harlan Laboratories. Heterozygous control mice were obtained by crossing pIV−/− K14 CIITA Tg with WT C57BL/6 mice. pIV^fl/fl^ vil-Cre Tg mice and pIV^fl/fl^ littermates were generated by crossing Vil-Cre-ER^T2^ mice [19] and pIV^fl/fl^ mice [17]. Rag1−/− IFN-γ−/− mice were obtained by crossing Rag1−/− mice [20] and IFN-γ−/− mice [21]. Mice were housed in specific-pathogen-free (SPF) facilities at the Universities of Lausanne or Bern. All experiments were approved by the institutional, Swiss federal and cantonal veterinary authorities (Permit number 1521.3), and all efforts were made to minimise suffering.

### Anti-IL-10R mAb-induced Colitis Model

Experimental mice were obtained from *H. hepaticus* infected parents. Prior and during *in vivo* experiments, the intestinal flora of mice was synchronized by repeatedly exchanging feces among experimental groups. Comparable *H. hepaticus* loads in individual mice were confirmed by qPCR (details in Methods S1). Mice were treated i.p. with 0.5 mg per injection of mAb 1B1.3a (anti–IL-10R), or isotype control mAb Y13-259 (anti-p21 Ras Epitope within amino acids 62–76), in PBS on days 0, 4, 7, 11, 14, 18, 21, and 25. For *in vivo* IFN-γ neutralization, mice were treated i.p. with 400 mg per injection of mAb XMG1.2 (anti-IFN-γ), or isotype control mAb Y13-259, in PBS on days 11, 14, 18, 21, 25, and 28. Weight of mice was followed daily during treatment until day 28. Mice were sacrificed for analysis 1 wk after the last anti-IL-10R mAb injection.

### CD4^+^ T-cell Transfer Colitis Model

Colitis was induced by adoptive transfer of 2×10^5^ CD4^+^ CD25^−^ CD45RB^hi^ FACS-sorted T cells from WT or IFN-γ−/− mice into Rag1−/− or Rag1−/− IFN-γ−/− mice. Animals were sacrificed at days 21–26 post CD4^+^ T cell transfer at the onset of severe clinical signs of colitis (diarrhoea, severe weight loss).

### Colon Histopathological Analysis

Intestinal tissues of the mid-colon were immediately frozen in Tissue-Tek O.C.T. compound (Sakura). 4–5 µm cross-sections were stained with hematoxylin and eosin, and inflammation was assessed blinded by a clinical pathologist on a scale of 0–15 according to the following criteria: Presence of lymphocyte infiltration in the mucosa (0–1 score), submucosa (0–1 score) and/or muscularis propia (0–1 score), cryptitis (0–3 score), ulceration (0–3 score), crypt erosion/destruction (0–3 score). The degree of inflammation was graded semi-quantitatively from 0 to 3 as follows: 0, no evidence of inflammation; 1, mild inflammation; 2, moderate inflammation; 3, severe inflammation. Microscopic images were acquired using a DFC295 camera connected to a DMIL LED light microscope via the FireCam Software (Leica Microsystems).

### Detection of Fecal Serum Albumin

Fresh feces were collected, lyophilized, and suspended in PBS. Fecal albumin levels were determined using the “Mouse albumin ELISA kit” (Bethyl Laboratories).

### Cell Preparations and Purifications

Single cell suspensions from colonic epithelial and lamina propria tissue fractions were obtained using a modification of an established protocol [22]. Briefly, longitudinally-cut colon samples were washed in PBS, further cut into small pieces, and incubated twice in Hank’s balanced-salt solution (HBSS) containing 5 mM EDTA and 2 mM DTT in a shaking incubator (37°C, 220 rpm) for 30 minutes to isolate cells from the intestinal epithelium. To derive lamina propria mononuclear cells, the tissue was further incubated in HBSS containing 0.1 U/mL collagenase D and 50 U/mL DNase I (both Roche) in a shaking incubator (37°C, 220 rpm) for 2–3 cycles of 30–40 minutes. After each incubation cycle, the collected fraction was sequentially filtered through a 70 µm and 40 µm cell strainer (BD Biosciences). For eventual further enrichment of lymphocytes via gradient centrifugation, derived cell fractions were resuspended in 40% Percoll (v/v) (GE Healthcare), layered on top of 80% Percoll (v/v) and centrifuged for 20 min at 1000×g at RT. Lymphocytes were recovered at the 40%/80% Percoll interphase. Single cell suspensions from lymphoid organs were obtained by mashing the organs through a 40 µm cell strainer.

### Flow Cytometry

Single cell suspensions were incubated with anti-FcγRII/III (2.4G2). The following antibodies (clones) were used for surface staining: anti-CD3ε (145-2C11), anti-CD4 (RM4-5), anti-CD8 (53-6.7), anti-CD11b (M1/70), anti-CD11c (N418), anti-CD25 (PC61.5), anti-CD40 (1C10), anti-CD44 (IM7), anti-CD45.2 (104), anti-CD62L (MEL-14), anti-CD90.2 (53-2.1), anti-Ly-6C (HK1.4), anti-Sca-1 (D7), anti-EpCAM (G8.8) (all from eBioscience), anti-CD80 (16-10A1), anti-CD86 (GL-1), anti-MHCII (M5/114.15.2), anti-Ly-6G (1A8) (all from Biolegend). Dead cells were excluded by eF506 viability staining. For transcription factor staining, cells were fixed and permeabilized using the transcription factor staining kit and were stained with anti-FoxP3 (FJK-16s) (both eBioscience). Samples were measured on an LSR II flow cytometer (BD Biosciences), and analysed via the FlowJo software (Tree Star).

### qPCR

Small colonic explants were shock frozen in liquid nitrogen before homogenisation using the Tissue Lyser II. RNA was isolated by the RNeasy mini kit (both Qiagen). cDNA was generated with the Superscript II reverse transcriptase kit (Life Technologies) and random nonamer primers. cDNA concentrations were adjusted and qPCR reactions were performed using the SYBR Green Master Mix (Roche) on a LightCycler 480 machine (Roche). The following primer sequences were used (from 5′ to 3′): *ccl3* forward CCAAGTCTTCTCAGCGCCAT, reverse TCCGGCTGTAGGAGAAGCAG; *ccl4* forward TCTTGCTCGTGGCTGCCT, reverse GGGAGGGTCAGAGCCCA; *ccl5* forward CCTCACCATCATCCTCACTGC, reverse TCTTCTCTGGGTTGGCACACA; *ifn-g* forward GGATGCATTCATGAGTATTG, reverse CTTTTCCGCTTCCTGAGG; *il-1b* forward CAACCAACAAGTGATATTCTCCATG, reverse GTGCCGTCTTTCATTACACAG; *il-6* forward GAGGATACCCTCCCAACAGACC, reverse AAGTGCATCATCGTTGTTCATACA; *Il-23p19* forward AGCGGGACATATGAATCTACTAAGAGA, reverse GTCCTAGTAGGGAGGTGTGAAGTTG; *t-bet* forward CAACAACCCCTTTGCCAAAG, and reverse TCCCCCAAGCAGTTGACAGT; *ror-γt* forward CCGCTGAGAGGGCTTCAC, and reverse TGCAGGAGTAGGCCACATTACA; *tbp* forward CCTTCACCAATGACTCCTATGAC, reverse CAAGTTTACAGCCAAGATTCAC; *b-actin* forward GCACAGCTTCTTTGCAGCTCCTTCG, reverse TTTGCACATGCCGGAGCCGTTG. Gene expression for each individual sample was normalised to the housekeeping genes TBP and β-actin via the qBASE PLUS software (Biogazelle).

### Detection of Secreted Cytokines in Colon Explants

Longitudinally-cut specimens of the mid-colon were rinsed with PBS and cultured for 6 h in IMDM +10% FCS +5×10^−5 ^M 2-ME at 37°C, 5% CO_2_. Debris was removed by centrifugation. IFN-γ (BD Biosciences) and IL-17A (eBioscience) were measured by ELISA. All other molecules were detected using the Mouse cytokine 20-Plex (Life Technologies) on a Luminex xMAP analyser (Merck Millipore). Concentrations were normalized to the weight of the colon explants.

### Statistical Analysis

Differences in weight gain between groups of mice were assessed by a repeated two-way analysis of variance (ANOVA), followed by a Bonferroni post-hoc test. For all other experiments, differences between groups were determined by the student’s unpaired t test or, when the normality test failed, by the Mann-Whitney Rank Sum test. *P* values are indicated when considered statistically significant (*P<0.05, **P<0.01, and ***P<0.001).

## Results

### Characterisation of T cells in pIV−/− K14 CIITA Tg Mice

It was previously shown that pIV−/− K14 CIITA Tg mice are deficient in nonhematopoietic MHCII expression but harbour normal thymic and peripheral CD4^+^ T cell populations displaying WT T cell receptor Vβ-chain repertoires [18]. Frequencies of CD4^+^ T cells, including FoxP3^+^ Treg cells, in colon-draining mesenteric and caudal lymph nodes, the colonic intestinal epithelium (cIE) and lamina propria (cLP) were found to be comparable between pIV−/− K14 CIITA Tg, heterozygous and WT mice (Figure S1). We also observed a normal distribution of CD4^+^ effector, memory, naïve and FoxP3^+^ T cell subsets in the thymus, spleen and peripheral lymph nodes (Figure S2). Finally, we examined CD4^+^ T cell-dependent B cell responses *in vivo* in pIV−/− K14 CIITA Tg mice upon immunisation with 4-Hydroxy-3-nitrophenylacetyl-hapten-23-conjugated chicken γ-globulin (NP_23_-CGG) or ovalbumin. Systemic antigen-specific antibody responses in pIV−/− K14 CIITA Tg mice were comparable to WT mice (Figure S3). In line, affinity maturation, assessed by detection of NP_4_-specific total serum IgG in NP_23_-CGG immunised mice, was similar to WT mice (Figure S3A). These results confirm that the phenotypes and functions of CD4^+^ T cells, including FoxP3^+^ Treg cells, in the colon and in primary and secondary lymphoid organs of pIV−/− K14 CIITA Tg mice are comparable to those of WT mice.

### Anti-IL-10R mAb Treatment during Chronic *H. hepaticus* Infection in the Absence of Inducible Nonhematopoietic MHCII Expression Leads to Colitis

To assess susceptibility to bacterial-driven intestinal inflammation, experimental mice chronically infected since birth with *H. hepaticus* were treated with anti-IL-10R mAb. Prior and during the treatment period, we synchronized the microbiome by repeatedly exchanging feces among mice, based on the recent observation that co-housed mice adopt each others’ microbial configuration in the fecal content [23], [24]. In addition, the low to undetectable colonic MHCII expression in the steady state (shown below) should not influence the flora. Notably, *H. hepaticus* loads were comparable in all experimental mice before and after anti-IL10R mAb treatment (Figure S4). It is important to mention that this experimental system is different from the previously published colitis model based on induction of chronic colitis by acute *H. hepaticus* infection [25]. In contrast, in our setting, chronically infected WT mice should not develop colitis.

**Figure 1:**
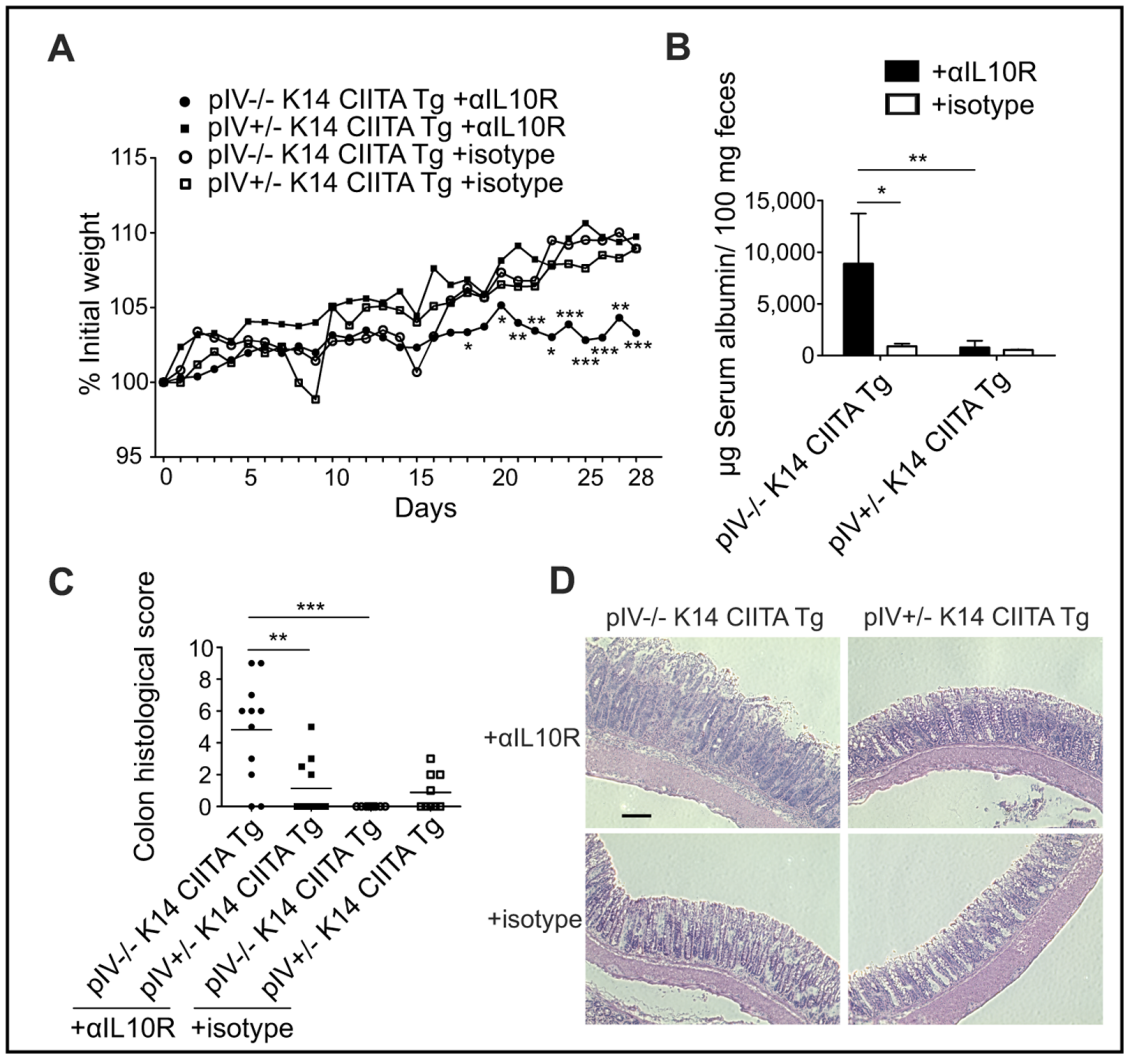
Chronic *H. hepaticus* infection plus anti-IL10R mAb treatment induces colitis in pIV−/− K14 CIITA Tg mice. (A) Development of body weight during anti-IL-10R mAb or isotype treatment of *H. hepaticus*-infected pIV−/− K14 CIITA Tg mice or pIV+/− K14 CIITA Tg controls (*n = *9–11 per group). (B) Serum albumin concentrations in feces collected on days 26–30 (*n = *6–8 per group). Data are shown as mean and s.d. and represent two pooled experiments. (C–D) Colon histopathological analysis on day 32; (C) colitis scores, data displayed as mean, and (D) representative photomicrographs of colon sections, stained with hematoxylin and eosin. Bar, 100 µm. Data represent three pooled experiments (*n = *9–11 per group). αIL10R, anti-interleukin-10 receptor monoclonal antibodies;

To evaluate whether the absence of inducible nonhematopoietic MHCII expression (shown below) alters susceptibility to bacterial-driven colitis, pIV−/− K14 CIITA Tg mice and heterozygous controls chronically infected since birth with *H. hepaticus* were treated with anti-IL-10R mAb. In contrast to MHCII-competent controls, pIV−/− K14 CIITA Tg mice gained significantly less weight (Figure 1A), displayed significantly elevated fecal serum albumin levels reflecting augmented protein-losing enteropathy (Figure 1B), and developed a mild but prominent diarrhoea that was not observed in heterozygous control mice (not shown). Histopathological examinations of colon sections revealed an exacerbated inflammation in pIV−/− K14 CIITA Tg mice (Figures 1C–D). pIV−/− K14 CIITA Tg mice exhibited a diffuse colonic mononuclear cell infiltration, whereas heterozygous controls displayed only focal infiltration (not shown).

We next used a conditional knockout system to determine whether the observed pathology is due to the lack of inducible MHCII expression on IECs. Mice in which pIV is flanked by loxP sites (pIV^fl/fl^) were crossed with mice harbouring a transgene in which expression of tamoxifen-inducible Cre-recombinase is controlled by an IEC-specific villin promoter (vil-Cre Tg). In pIV^fl/fl^ vil-Cre Tg mice, tamoxifen induces pIV excision exclusively in IECs. Upon the administration of tamoxifen and anti-IL-10R mAb pIV^fl/fl^ vil-Cre Tg mice displayed increased colitis susceptibility (Figures S5A–C), indicating that the absence of inducible MHCII expression specifically on IECs significantly exacerbates chronic *H. hepaticus*-mediated colitis. However, we noted that tamoxifen altered the homeostatic composition of gut lymphocytes (not shown) and affected colitis in control mice. This is consistent with the fact that tamoxifen substantially affects the murine gastrointestinal tract [26], [27]. Due to the adverse effects of tamoxifen we focussed on the constitutively pIV-depleted mice for further experiments.

### Colitic pIV−/− K14 CIITA Tg Mice Display Increased Innate Effector Cell Infiltration and Elevated Expression of Proinflammatory Chemokines and Cytokines

**Figure 2:**
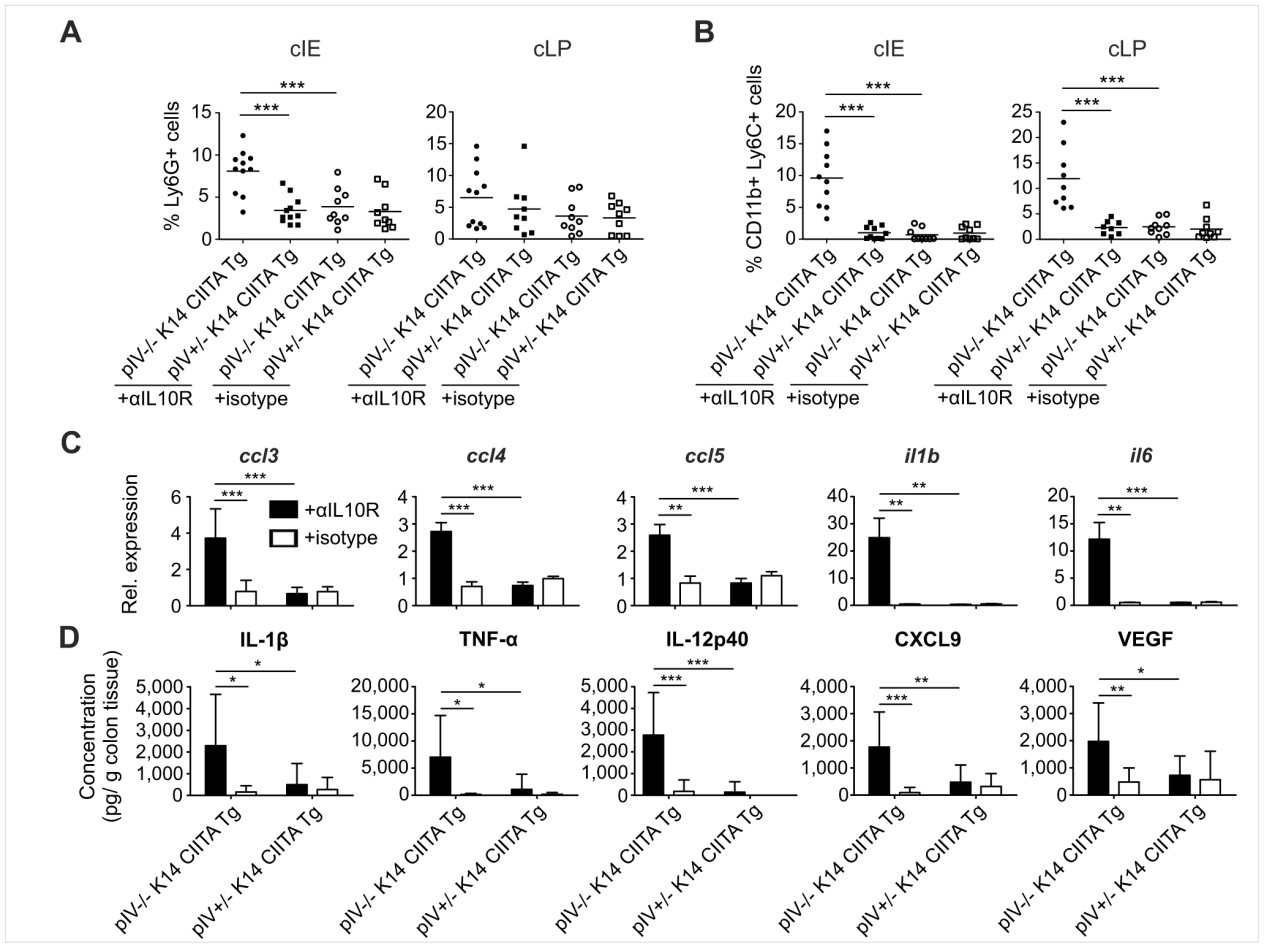
Innate effector cells and proinflammatory cytokines are elevated in colitic pIV−/− K14 CIITA Tg mice. (A–B) Frequency of Ly6G^+^ neutrophil granulocytes (A) and CD11b^+^ Ly6C^+^ inflammatory monocytes (B) isolated from the colonic intestinal epithelium (cIE, left panel) and the colonic lamina propria (cLP, right panel) of *H. hepaticus*-infected pIV−/− K14 CIITA Tg mice or pIV+/− K14 CIITA Tg controls. (C) *ccl3*, *ccl4*, *ccl5*, *il1b* and *il6* mRNA expression levels in colon explants. (D) IL-1β, TNF-α, IL-12p40, CXCL9 and VEGF secretion upon *ex vivo* organ culture of colon explants. All data represent three pooled experiments (*n = *9–11 per group). αIL10R, anti-interleukin-10 receptor monoclonal antibodies; IL, interleukin; TNF, tumor necrosis factor; VEGF, vascular endothelial growth factor;

We next sought to identify the major cellular and molecular players mediating exacerbated inflammation and tissue damage in colitic pIV−/− K14 CIITA Tg mice. Infiltration of Ly6G^+^ neutrophils was increased in the colons of colitic pIV−/− K14 CIITA Tg mice, which was significant in the cIE but not the cLP (Figure 2A). CD11b^+^ Ly6C^+^ inflammatory monocytes were significantly elevated in both the cIE and cLP (Figure 2B). These innate effector cell subsets are also augmented in IBD patients [2]. We also noted a mild increase in CD11c^+^ DCs in the inflamed colons (Figure S6). mRNA quantification revealed a significant elevation of mRNAs encoding the proinflammatory chemokines CCL3, CCL4 and CCL5 in the colons of colitic pIV−/− K14 CIITA Tg mice (Figure 2C). These chemokines recruit innate effector cells and T cells to sites of inflammation [28]. Moreover, mRNAs encoding the inflammatory cytokines IL-1β and IL-6 were significantly increased (Figure 2C). We also found enhanced colonic secretion of proinflammatory IL-1β, tumour necrosis factor (TNF)-α and IL-12p40, as well as CXCL9 and vascular endothelial growth factor (VEGF) in colitic pIV−/− K14 CIITA Tg mice (Figure 2D). Increased expression of IL-1β, TNF-α, IL-6 and IL-12p40 was previously observed in IBD patients [1], [2]. CXCL9 secreted by innate effector cells was shown to promote T cell activation and recruitment [29]. VEGF is known to increase vascular permeability and inflammatory leukocyte extravasation in IBD and experimental colitis [30]. In summary, the cellular and molecular mediators of intestinal inflammation in colitic pIV−/− K14 CIITA Tg mice display similarities to those in IBD, underlining the physiological relevance of the applied colitis model.

### Exacerbated Colitis in pIV−/− K14 CIITA Tg Mice Correlates with the Inability of IECs to Upregulate MHCII Expression

**Figure 3:**
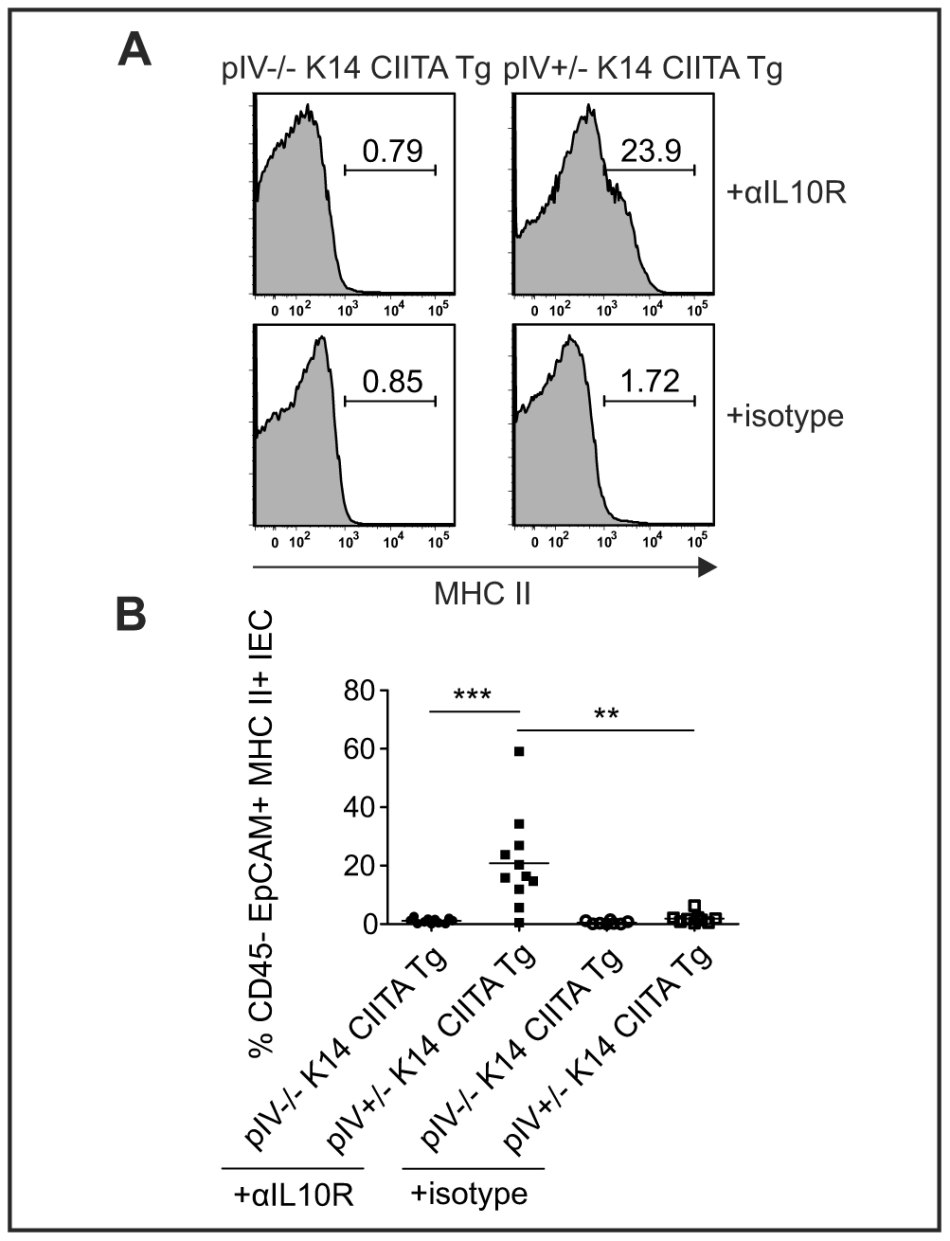
Colitic pIV−/− K14 CIITA Tg mice lack inducible MHCII expression by colonic IECs. (A–B) Frequency of CD45.2^−^ EpCAM^+^ MHCII^+^ IECs isolated from anti-IL-10R mAb or isotype treated, *H. hepaticus*-infected pIV−/− K14 CIITA Tg mice or pIV+/− K14 CIITA Tg controls. Representative histograms (A) and summarized data as mean (B) from three pooled experiments (*n = *8–11 per group). αIL10R, anti-interleukin-10 receptor monoclonal antibodies; IEC, intestinal epithelial cell;

We next examined whether exacerbated colitis in pIV−/− K14 CIITA Tg mice correlates with the loss of inducible MHCII expression by IECs. IECs in anti-IL-10R-treated heterozygous control mice upregulated MHCII expression, while this was not observed in colitic pIV−/− K14 CIITA Tg mice or healthy isotype-treated controls (Figure 3). The same was true in the inducible IEC-specific MHCII knock-out system (Figure S5D). Confocal microscopy revealed that IECs from anti-IL-10R-treated heterozygous mice expressed MHCII molecules on the basolateral and apical surface (Figure S7). Since the provision of costimulation by APCs is a prerequisite for immunogenic T cell activation, we examined whether IECs express costimulatory molecules in healthy and/or colitic mice. We did not detect induction of the classical costimulatory molecules CD40, CD80 and CD86 (Figure S8). Collectively, these results confirm that exacerbated colitis in pIV−/− K14 CIITA Tg mice correlates with the inability of IECs to express MHCII.

### Colitic pIV−/− K14 CIITA Tg Mice Display Elevated Frequencies of Colonic CD4^+^ Th1 Cells and an Increased CD4^+^ T cell: FoxP3^+^ Treg Cell Ratio

**Figure 4:**
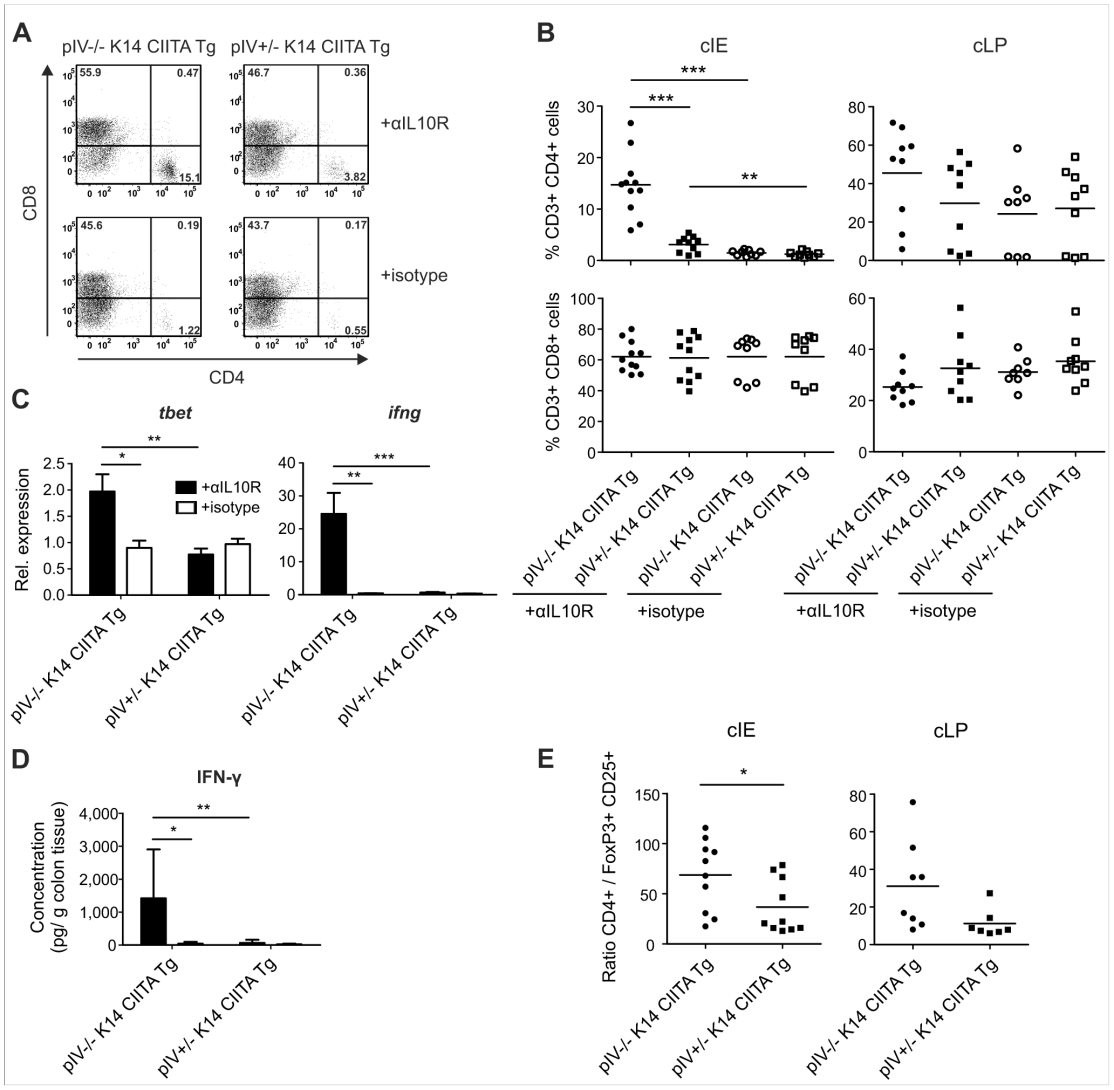
Colitic pIV−/− K14 CIITA Tg mice display elevated Th1 cells, IFN-γ, and CD4^+^ T cell: FoxP3^+^ Treg cell ratios. (A–B) Frequency of CD3^+^ CD4^+^ and CD8^+^ T cells isolated from anti-IL-10R mAb or isotype treated, *H. hepaticus*-infected pIV−/− K14 CIITA Tg mice or pIV+/− K14 CIITA Tg controls. Representative histograms from the colonic intestinal epithelium (cIE) (A) and summarized data (B) from cIE (left) and the colonic lamina propria (cLP) (right) as mean. (C) *ifng* and *tbet* mRNA expression levels in colon explants. (D) IFN-γ secretion upon *ex vivo* organ culture of colon explants as means and s.d. (A–D) Data represent three pooled experiments (*n = *9–11 per group). (E) Ratio of absolute numbers of CD4^+^ T cells:absolute numbers of CD25^+^ FoxP3^+^ Treg cells from cIE (left) and cLP (right) as mean from two pooled experiments (*n = *7–10 per group). αIL10R, anti-interleukin-10 receptor monoclonal antibodies; FoxP3, forkhead box P3; IFN, interferon;

We next investigated the impact of deficient epithelial MHCII expression on intestinal T cells during colitis. We observed an increase in colonic CD4^+^ but not CD8^+^ T cells in colitic pIV−/− K14 CIITA Tg mice, which was significant in the cIE but not the cLP (Figures 4A–B). Colitic heterozygous mice only displayed a mild elevation of CD4^+^ T cells. Similarly, CD4^+^ T cells were significantly elevated in colitic pIV^fl/fl^ vil-Cre Tg mice, which lack MHCII expression specifically on IECs (Figure S5E). Examination of T cell polarization revealed significantly augmented expression of mRNAs encoding the Th1 signature factors T-bet and IFN-γ, as well as IFN-γ secretion, in inflamed colons of pIV−/− K14 CIITA Tg mice (Figures 4C–D).

Natural cytotoxicity-triggering receptor (NCR^−^) group 3 innate lymphoid cells (ILC) cells were recently identified as important cellular mediators of *H. hepaticus*-driven innate colitis [31], [32]. However, we did neither detect a specific increase in these cells (Figure S6B) nor in the expression of *ror-γt*, *il23p19* or IL-17A, which are diagnostic for group 3 ILCs and Th17 responses (Figure S9). Thus, although we do not exclude a contribution of these cells to the inflammatory process, they are unlikely to play a dominant role in the increased pathology observed in our system.

Interestingly, we observed that colonic CD4^+^ T cells from colitic pIV−/− K14 CIITA Tg mice displayed a mildly reduced expression of the co-inhibitory marker programmed cell death (PD)-1 when compared to heterozygous mice (not shown). We also found a significantly increased CD4^+^ T cell: FoxP3^+^ Treg cell ratio in the cIE of colitic pIV−/− K14 CIITA Tg mice compared to identically treated heterozygous control mice (Figure 4E). The majority of FoxP3^+^ cells lacked neuropillin (Nrp)-1 expression in both anti-IL-10R-treated groups (not shown), suggesting that the majority of colonic Treg cells were generated peripherally [33]. Taken together, these results indicate that MHCII expression by IECs attenuates bacterial-driven colitis by preventing exacerbated effector Th1 cell accumulation and the establishment of an unfavourably altered ratio between conventional CD4^+^ T cells and FoxP3^+^ Treg cells.

### Epithelial MHCII Expression is Induced by IFN-γ Mainly Derived from CD4^+^ T cells

**Figure 5:**
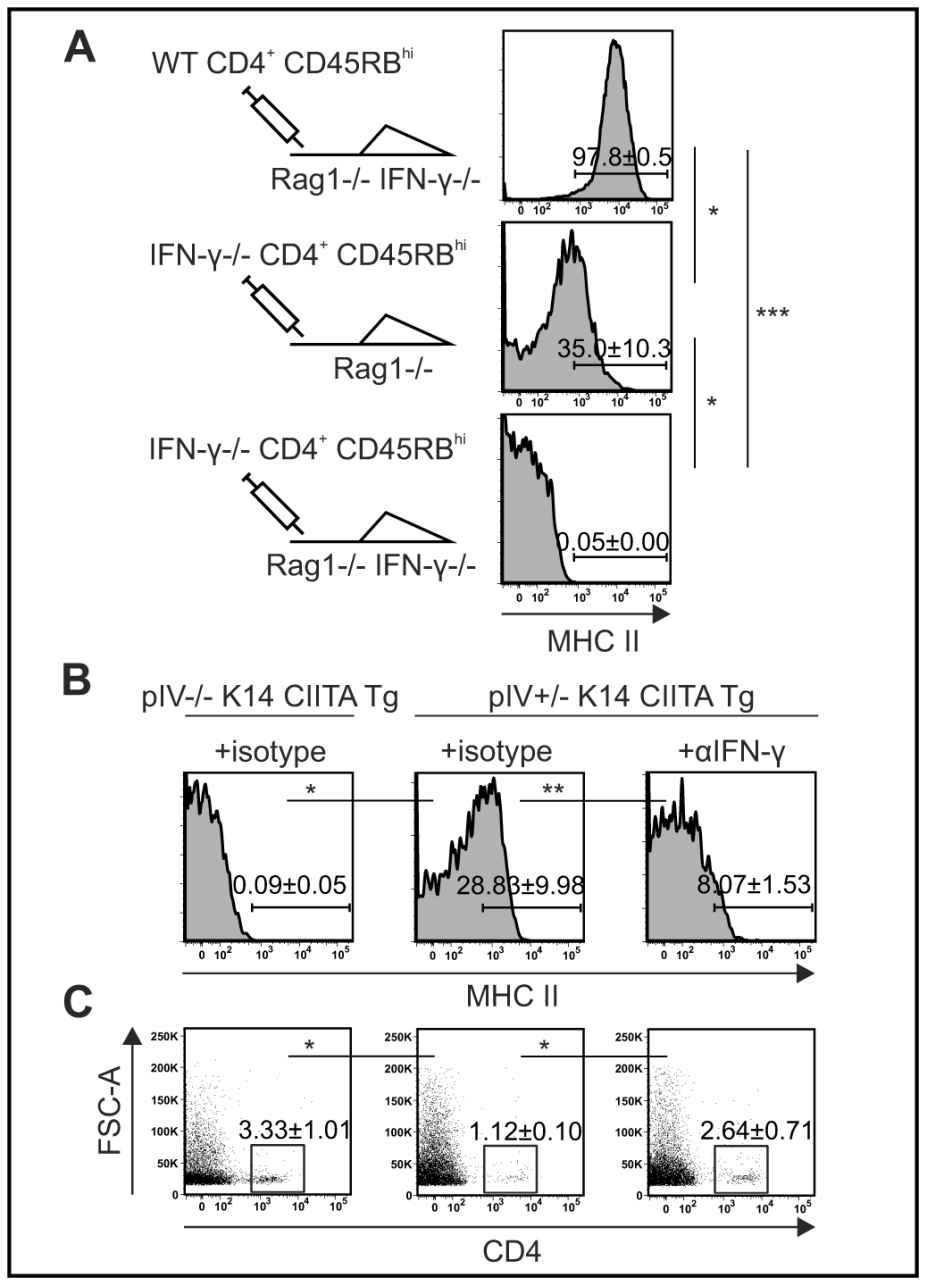
IFN-γ induces MHCII expression on IECs. (A) Frequency of CD45.2^−^ EpCAM^+^ MHCII^+^ intestinal epithelial cells (IEC) isolated from Rag1−/− or Rag1−/− IFN-γ−/− mice that were adoptively transferred with CD4^+^ CD45RB^hi^ T cells from WT or IFN-γ−/− mice shown as means and SEM in representative histograms (*n* = 3 mice per group). (B–C) Frequency of CD45.2^−^ EpCAM^+^ MHCII^+^ IECs (B) and CD4^+^ T cells from the colonic intestinal epithelium (C) isolated from *H. hepaticus*-infected, anti-IL-10R mAb-administered pIV−/− K14 CIITA Tg, pIV+/− K14 CIITA Tg or pIV+/− K14 CIITA Tg that were treated with neutralizing anti-IFN-γ mAb. Shown are representative FACS plots, means and SEM from two pooled experiments (*n* = 4–7 mice per group). αIFN-y, anti-interferon-γ monoclonal antibodies; FSC, forward scatter; IFN, interferon; Rag, recombination activating gene; WT, wild type;

To examine whether IFN-γ is responsible for inducing epithelial MHCII expression we made use of the adoptive transfer colitis model in which lymphocyte-deficient mice develop colitis upon transfer of CD4^+^ CD45RB^hi^ T cells [34]. Transfer of WT CD4^+^ T cells into Rag1−/− IFN-γ−/− mice resulted in prominent upregulation of epithelial MHCII expression, which was markedly reduced when IFN-γ−/− CD4^+^ T cells were transferred into IFN-γ-competent Rag1−/− mice (Figure 5A). Notably, IECs did not upregulate MHCII expression when both donor and recipient mice lacked IFN-γ (Figure 5A). Importantly, all experimental groups developed severe colitis as assessed by histopathology (Brasseit *et al*., manuscript in preparation). These results indicate that IFN-γ is the major cytokine driving MHCII expression on IECs during adoptive transfer colitis, and that CD4^+^ T cells represent the major source of IFN-γ in this process.

To evaluate whether IFN-γ-induced epithelial MHCII expression protects against colitis we administered neutralizing anti-IFN-γ mAb to pIV+/− K14 CIITA Tg mice 11 days after initial anti-IL-10R treatment to avoid interference with T cell polarization. Neutralization of IFN-γ in anti-IL-10R-treated pIV+/− K14 CIITA Tg mice reduced MHCII expression by IECs as compared to isotype-treated mice (Figure 5B). Importantly, this resulted in elevated frequencies of CD4^+^ T cells in the cIE of anti-IFN-γ mAb-treated mice, comparable to pIV−/− K14 CIITA Tg mice (Figure 5C). In addition, preliminary data indicates that the CD4^+^ T cell: FoxP3^+^ Treg cell ratio is mildly elevated in anti-IFN-γ-administered pIV+/− K14 CIITA Tg mice compared to isotype-treated mice (not shown). Collectively, these findings suggest that IFN-γ-mediated MHCII expression by IECs plays an anti-inflammatory role by reducing the accumulation of colitogenic CD4^+^ T cells during chronic bacterial-driven colitis.

## Discussion

We report here that the abrogation of inducible MHCII expression on IECs during chronic *H. hepaticus* infection and anti-IL-10R mAb treatment leads to overt colitis associated with an augmented accumulation of CD4^+^ Th1 cells and an increased CD4^+^ T cell:FoxP3^+^ Treg cell ratio. In contrast, heterozygous control mice do not develop intestinal pathology as assessed by clinical parameters, despite the fact that they display increased MHCII expression by IECs and exhibit mildly elevated levels of infiltrating CD4^+^ T cells. Finally, we show that epithelial MHCII expression is induced by IFN-γ produced mainly by CD4^+^ T cells.

We also examined whether NCR^−^ group 3 ILCs - which were recently described to have a major role in murine innate colitis [31], [32] - or Th17 cells might be responsible for promoting intestinal inflammation in the absence of nonhematopoietic MHCII. However, we observed comparable group 3 ILC frequencies in all anti-IL-10R mAb-administered animal groups, and no increase in associated factors ROR-γt, IL-23 and IL-17A. Thus, albeit group 3 ILCs and Th17 cells may play an important role in colitis, we exclude a decisive role in determining disease severity in our model.

Induction of MHCII on IECs correlated with a protection against colitis in heterozygous mice, indicating that the low-levels of IFN-γ observed in heterozygous mice were sufficient to maintain intestinal homeostasis via the upregulation of MHCII expression during chronic *H. hepaticus* infection and anti-IL-10R treatment. IECs are well known to fulfil diverse functions crucial for intestinal homeostasis, including the capacity to modulate intestinal immune responses [3]. Here we expand these previous findings by reporting an MHCII-dependent anti-inflammatory role of IECs that confers protection against colitis induced by chronic *H. hepaticus* colonization.

Previous studies examining potential antigen-presentation functions of IECs and their consequences for intestinal inflammation generated conflicting results and relied almost exclusively on *in vitro* or *ex vivo* assays [14], [15], [16]. One recent *in vivo* study using a mouse model in which MHCII is expressed solely on IECs indicated that antigen presentation by bone marrow-derived APCs is indispensable for triggering severe bacterial-driven intestinal pathology [35].

Immunogenic T cell activation requires TCR-MHCII-dependent signals, the presence of polarizing cytokines and the provision of costimulation. Whether IECs can express classical costimulatory molecules remains a matter of debate [11], [12], [13]. In our model we did not observe expression of the classical costimulatory molecules CD40, CD80 or CD86 on MHCII^+^ IECs. Antigen-presentation in the absence of costimulatory molecules has been proposed to limit CD4^+^ T cell responses [36], which is consistent with the finding that IECs are not *bona fide* professional APCs capable of promoting severe colitis *in vivo* [35]. However, non-classical costimulatory molecules, such as IcosL, PD-L1 or LFA3 have been proposed to be used by IECs to interact with CD4^+^ T cells [12], [37].

The anti-inflammatory cytokine IL-10 is critical for maintaining local tissue homeostasis in the presence of intestinal *H. hepaticus* infection. First indications for this came from early observations in IL-10-deficient mice that developed spontaneous colitis under conventional housing conditions which was significantly less severe (or even absent) when mice were held under SPF conditions [38]. Deliberate infection of SPF-housed IL-10−/− mice with *H. hepaticus* significantly exacerbated the development of colitis [39], and susceptibility to colitis induction is re-established in WT mice, treated with anti-IL-10R mAb following *H. hepaticus* infection [25]. Furthermore, *H. hepaticus* was shown to induce colitis in lymphocyte-deficient 129/SvEv Rag2−/− mice [32], [40], [41]. However, adoptive transfer of Treg cells inhibits the development of gut inflammation in this colitis model [41], [42], [43], which depends on the ability of transferred cells to express IL-10 [43], [44]. Similarly, IL-10 is important to prevent colitis in humans, as certain variants of early-onset IBD observed in infants and small children appear to be a monogenic diseases caused by deleting mutations in IL-10 or its receptor (reviewed in [45]).

In contrast, the complex role of IFN-γ during *H. hepaticus*-induced colitis is incompletely understood. Injection of IFN-γ-neutralizing mAb into *H. hepaticus*-infected IL-10−/− mice suggested that IFN-γ is required for disease onset but not for the chronicity of colitis [39], [46]. Similarly, *H. hepaticus*-infected IFN-γ−/− mice treated with anti-IL-10R mAb developed less intestinal inflammation than WT mice [25], suggesting a contribution of IFN-γ to colitis development. In contrast, the severity of *H. hepaticus*-induced colitis in mice lacking both IL-10 and IFN-γ was comparable to mice lacking IL-10 alone [46], indicating that IFN-γ does not favour colitis development. Recently, it was reported that during the course of *H. hepaticus*-mediated colitis, induced Th17 cells switch phenotype to become IFN-γ^+^ ex-Th17 cells [47]. These results imply A) the potential existence of alternative pathways affecting colitis development following acute *H. hepaticus* infection, e.g. via the increase of inflammatory Th17 cells in the absence of IFN-γ, and B) that IFN-γ may have both pro- and anti-inflammatory effects on the outcome of *H. hepaticus*-mediated colitis [25]. Our results expand these previous observations by demonstrating that IFN-γ-mediated upregulation of MHCII molecules on IECs plays an anti-inflammatory role that reduces infiltrating CD4^+^ effector T cell frequencies and avoids the establishment of a pathologically altered CD4^+^ T cell:FoxP3^+^ Treg cell ratio in the colon. Of note, IFN-γ was reported to feature anti-inflammatory properties in the context of oral tolerance [48].

Our findings may help to explain why anti-IFN-γ treatments have consistently failed to induce remission in patients with active IBD despite the association of enhanced IFN-γ expression with IBD [49]. Moreover, this study paves the way for further work on the MHCII-dependent tolerogenic function of IECs as a potential therapeutic target in patients suffering from inflammatory disorders of the intestine.

## Supporting Information

Figure S1 **Intestinal FoxP3^+^ Treg cell frequencies in healthy pIV−/− K14 CIITA Tg mice.** (A–D) Healthy pIV−/− K14 CIITA Tg, pIV+/− K14 CIITA Tg and C57BL/6 WT mice were subjected to flow cytometry. (A) Mesenteric lymph node (mLN), (B) caudal lymph node (cLN), (C) colonic intestinal epithelium (cIE) and (D) colonic lamina propria (cLP) cells were gated on CD45.2^+^ CD4^+^ cells, and from there on FoxP3^+^ cells. Data shown represents mean and s.d. (*n = *3 per group). FoxP3, forkhead box P3; WT, wild type;
(TIF)

Figure S2 **Lymphoid organ T cell frequencies in healthy pIV−/− K14 CIITA Tg mice.** (A–C) Thymus (THY), spleen (SPL) and pooled peripheral lymph nodes (LN) of healthy pIV−/− K14 CIITA Tg, pIV+/− K14 CIITA Tg and C57/BL6 wild type (WT) mice were subjected to flow cytometry. Dead cells were excluded and CD3^+^ cells were gated on (A) CD4 and CD8. (B) CD4^+^ CD8^−^ T cells were gated on CD44 and CD62L to identify effector (T_eff_, CD44^+^ CD62L^−^), memory (T_mem_, CD44^+^ CD62L^+^) and naïve T (T_naive_, CD44^−^ CD62L^+^) cells. (C) CD4^+^ T cells were gated on CD25 and FoxP3. Data shown represents mean and s.d. (*n = *3 per group) from one experiment out of at least two experiments.
(TIF)

Figure S3 **Similar specific total IgG responses upon exogenous antigen immunisation in pIV−/− K14 CIITA Tg mice.** (A–B) pIV−/− K14 CIITA Tg, pIV+/− K14 CIITA Tg and B6 WT mice were immunised with 4-Hydroxy-3-nitrophenylacetyl hapten-conjugated chicken gamma globulin (NP_23_-CGG) or ovalbumin. Serum was analysed for the presence of antigen-specific total IgG against either (A) NP_4_ and (B) ovalbumin. Data represent mean and s.d. (*n = *7–8 per group) from two pooled experiments. IgG, immunoglobulin G;
(TIF)

Figure S4 ***H. hepaticus*** **colonization levels before and after anti-IL-10R treatment.** Fresh fecal specimens from pIV−/− K14 CIITA Tg and pIV+/− K14 CIITA Tg mice were collected on days −4 to −2 before anti-IL-10R administration, and on days 26–28 of the experiment. Total fecal DNA was isolated and *H. hepaticus* DNA was quantified by qPCR and normalized to the dry weight of the fecal pellet. Each symbol represents a single animal. αIL10R, anti-interleukin-10 receptor monoclonal antibodies;
(TIF)

Figure S5 ***H. hepaticus*** **infection plus anti-IL10R mAb treatment induces exacerbated colitis in pIV^fl/fl^ vil-Cre Tg mice.** (A) Development of body weight during anti-IL-10R mAb or isotype treatment of *H. hepaticus*-infected, tamoxifen-administered pIV^fl/fl^ vil-Cre Tg mice or pIV^fl/fl^ controls. Data are shown as mean. (B) Serum albumin concentrations in feces collected on days 26–30. Data are shown as mean and s.d. (C) Colitis scores upon organ collection on day 32. Data displayed as mean. (D) Frequency of CD45.2^−^ EpCAM^+^ MHCII^+^ IECs. Representative histograms (left) and summarized data (right) as mean. (E) Frequency of CD3^+^ CD4^+^ and CD8^+^ T cells in the cIE. Representative histograms (left) and summarized data (right) as mean. All data represent *n = *3–5 per group. αIL10R, anti-interleukin-10 receptor monoclonal antibodies;
(TIF)

Figure S6 **Levels of colonic DC and group 3 ILCs in colitic pIV−/− K14 CIITA Tg.** (A) Frequency of Ly6C^−^ CD11c^+^ conventional DCs in the cIE and cLP and (B) CD45.2^+^ Lin^−^ (CD11b, Gr-1, B220) CD3ε^−^ Thy1^high^ Sca-1^+^ group 3 ILCs in the cLP isolated from anti-IL-10R mAb or isotype treated, *H. hepaticus*-infected pIV−/− K14 CIITA Tg mice or pIV+/− K14 CIITA Tg controls. (A) Representative histograms (left) display the cIE and data (right) represent mean (*n = *9–11 per group) from three pooled experiments. (B) dot plots showed represent *n = *3–4 per group. αIL10R, anti-interleukin-10 receptor monoclonal antibodies;
(TIF)

Figure S7 **Colonic IECs from anti-IL-10R-treated pIV+/− K14 CIITA Tg mice express MHCII molecules apically and basolaterally.** (A and B) *H. hepaticus*-infected pIV−/− K14 CIITA Tg or pIV+/− K14 CIITA Tg control mice were treated with anti-IL-10R mAb or isotype control mAb. Mid-colon sections were stained with DAPI (blue) to label nuclei and anti-MHCII mAb (red). (A) Representative pIV−/− K14 CIITA Tg mouse and (B) representative pIV+/− K14 CIITA Tg mouse. Right upper panel depicts a region with MHCII^−^ IECs; Right lower panel depicts a region with MHCII^+^ IECs. Bar, 20 µm.
(TIF)

Figure S8 **Colitic mice do not induce the expression of CD40, CD80 and CD86 on colonic IECs.** (A–C) CD45.2^−^ EpCAM^+^ IECs isolated from anti-IL-10R mAb or isotype treated, *H. hepaticus*-infected pIV−/− K14 CIITA Tg mice or pIV+/− K14 CIITA Tg controls were analysed for the expression of classical costimulatory molecules by flow cytometry. (A) Frequency of CD40, (B) CD80 or (C) CD86. Histograms represent *n = *3–5 per group. αIL10R, anti-interleukin-10 receptor monoclonal antibodies;
(TIF)

Figure S9 **Expression levels of Th17- and group 3 ILC-associated factors.** (A–B) *H. hepaticus*-infected pIV−/− K14 CIITA Tg or pIV+/− K14 CIITA Tg control mice were treated with anti-IL-10R mAb or isotype control mAb. (A) *ror-γt* and *il23p19* mRNA expression levels in colon explants. Data represent *n = *9–11 per group from three pooled experiments. (B) IL-17A secretion upon *ex vivo* organ culture of colon explants. Data represent *n = *6 per group from two pooled experiments. Data displayed as mean and s.d. αIL10R, anti-interleukin-10 receptor monoclonal antibodies;
(TIF)

Methods S1 **Additional methods applied to generate supporting figures.**
(DOC)
