# Supplementary figures and images for: Interferon-γ Induces Expression of MHC Class II on Intestinal Epithelial Cells and Protects Mice from Colitis
Source: PLoS One. 2014 Jan 28;9(1):e86844. doi: 10.1371/journal.pone.0086844 (PMC3904943; doi:10.1371/journal.pone.0086844)

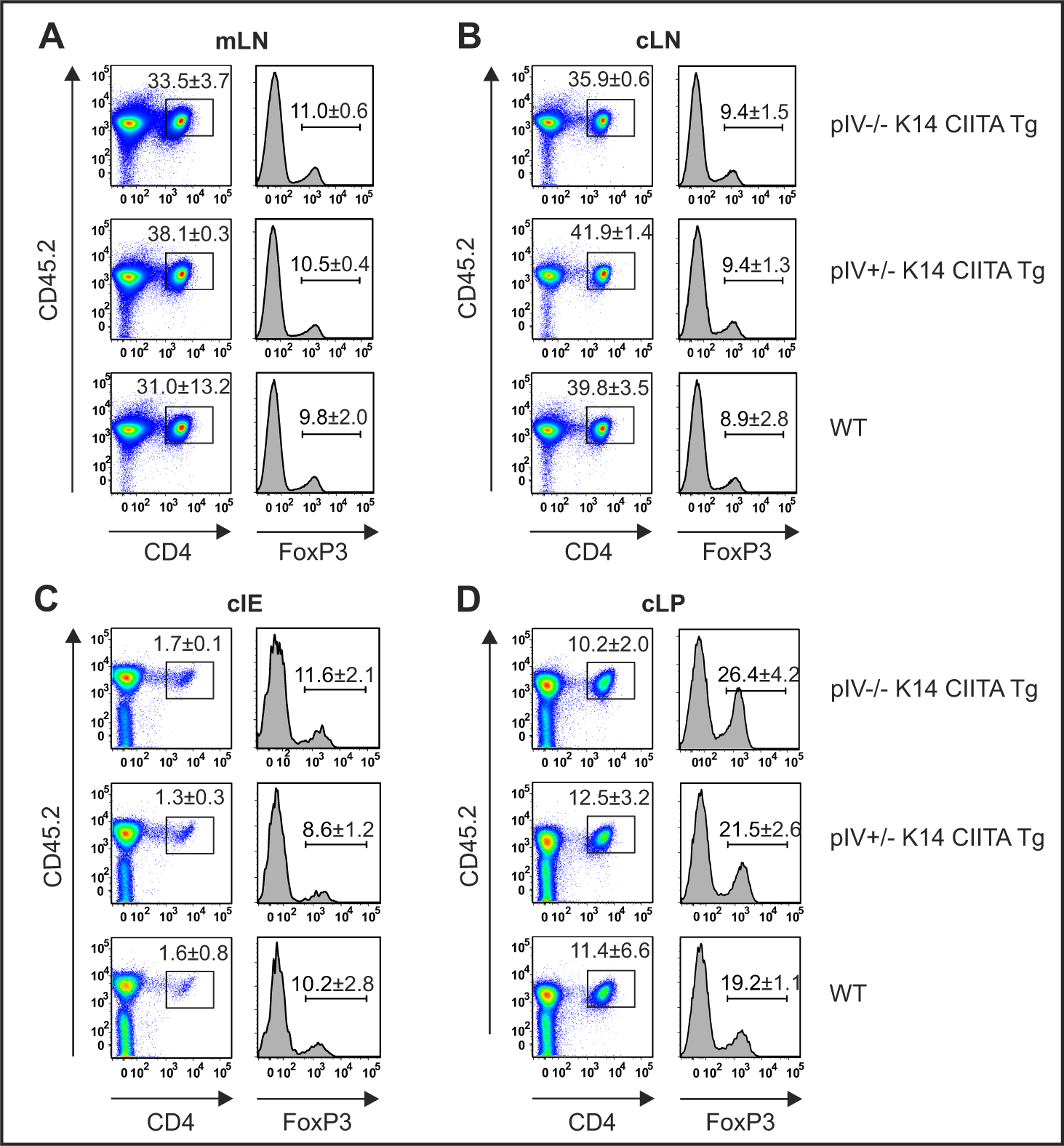

Supplement: Figure S1 — Intestinal FoxP3+ Treg cell frequencies in healthy pIV−/− K14 CIITA Tg mice. (A–D) Healthy pIV−/− K14 CIITA Tg, pIV+/− K14 CIITA Tg and C57BL/6 WT mice were subjected to flow cytometry. (A) Mesenteric lymph node (mLN), (B) caudal lymph node (cLN), (C) colonic intestinal epithelium (cIE) and (D) colonic lamina propria (cLP) cells were gated on CD45.2+ CD4+ cells, and from there on FoxP3+ cells. Data shown represents mean and s.d. (n = 3 per group). FoxP3, forkhead box P3; WT, wild type; (TIF) [file pone.0086844.s001.tif]

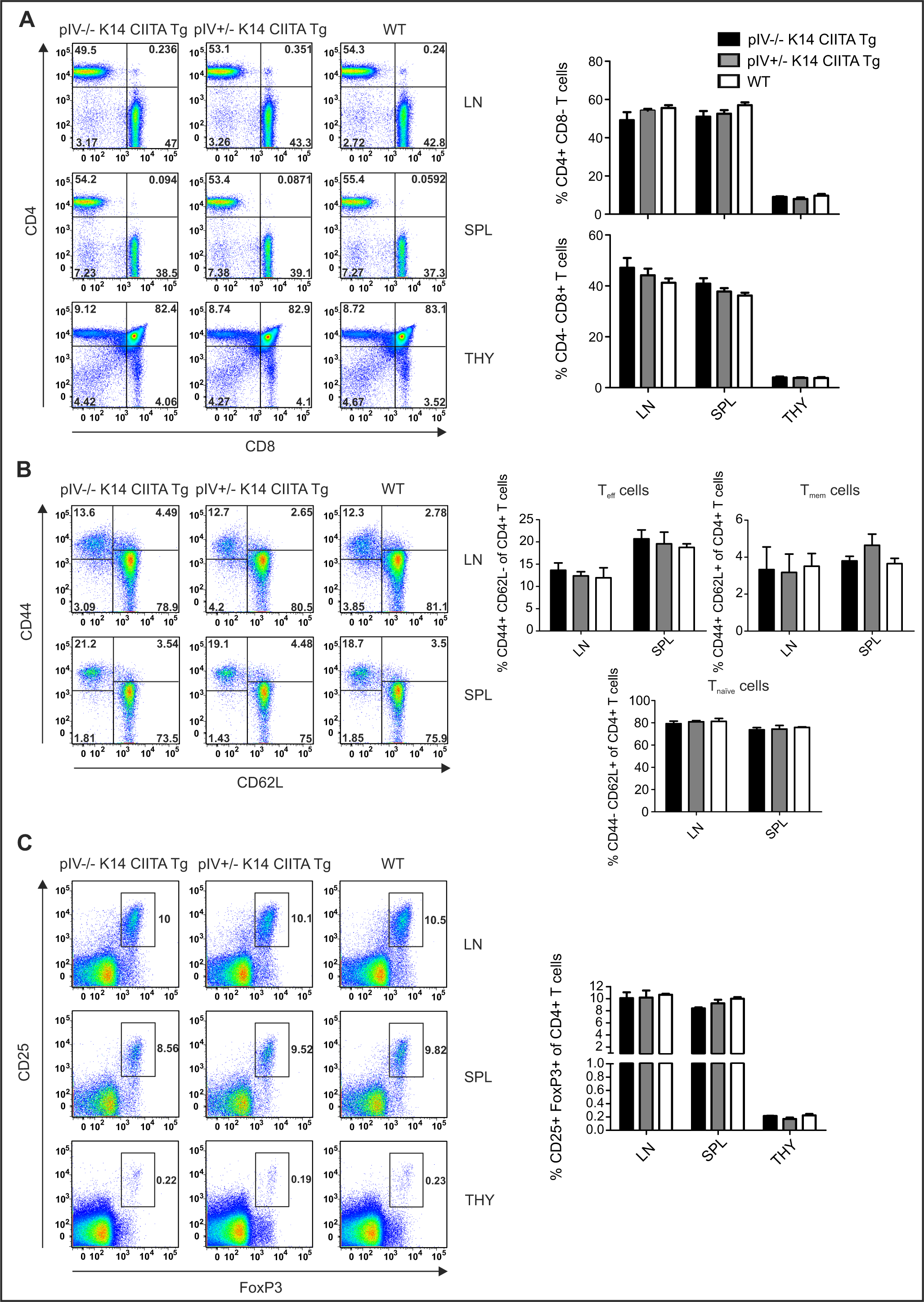

Supplement: Figure S2 — Lymphoid organ T cell frequencies in healthy pIV−/− K14 CIITA Tg mice. (A–C) Thymus (THY), spleen (SPL) and pooled peripheral lymph nodes (LN) of healthy pIV−/− K14 CIITA Tg, pIV+/− K14 CIITA Tg and C57/BL6 wild type (WT) mice were subjected to flow cytometry. Dead cells were excluded and CD3+ cells were gated on (A) CD4 and CD8. (B) CD4+ CD8− T cells were gated on CD44 and CD62L to identify effector (Teff, CD44+ CD62L−), memory (Tmem, CD44+ CD62L+) and naïve T (Tnaive, CD44− CD62L+) cells. (C) CD4+ T cells were gated on CD25 and FoxP3. Data shown represents mean and s.d. (n = 3 per group) from one experiment out of at least two experiments. (TIF) [file pone.0086844.s002.tif]

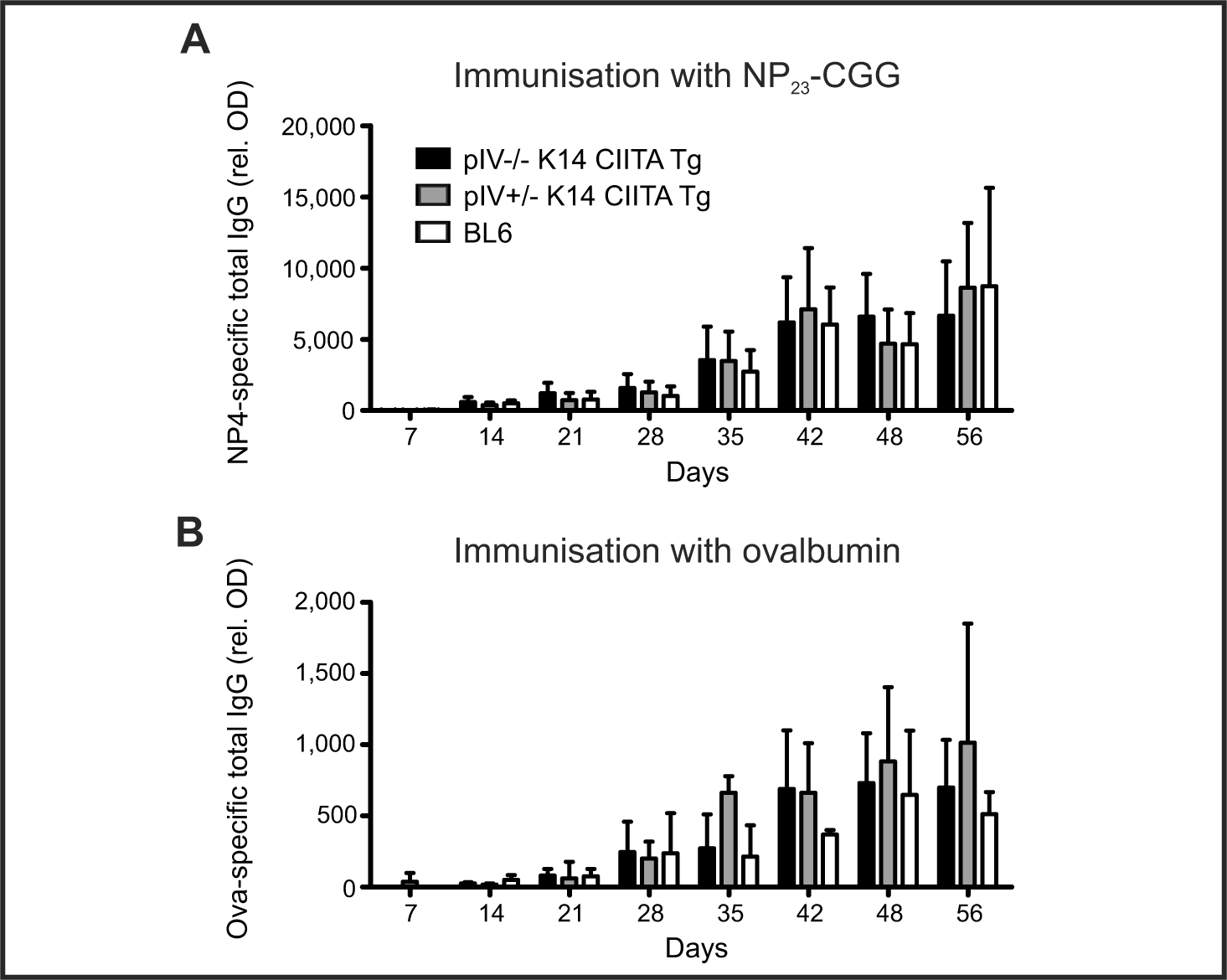

Supplement: Figure S3 — Similar specific total IgG responses upon exogenous antigen immunisation in pIV−/− K14 CIITA Tg mice. (A–B) pIV−/− K14 CIITA Tg, pIV+/− K14 CIITA Tg and B6 WT mice were immunised with 4-Hydroxy-3-nitrophenylacetyl hapten-conjugated chicken gamma globulin (NP23-CGG) or ovalbumin. Serum was analysed for the presence of antigen-specific total IgG against either (A) NP4 and (B) ovalbumin. Data represent mean and s.d. (n = 7–8 per group) from two pooled experiments. IgG, immunoglobulin G; (TIF) [file pone.0086844.s003.tif]

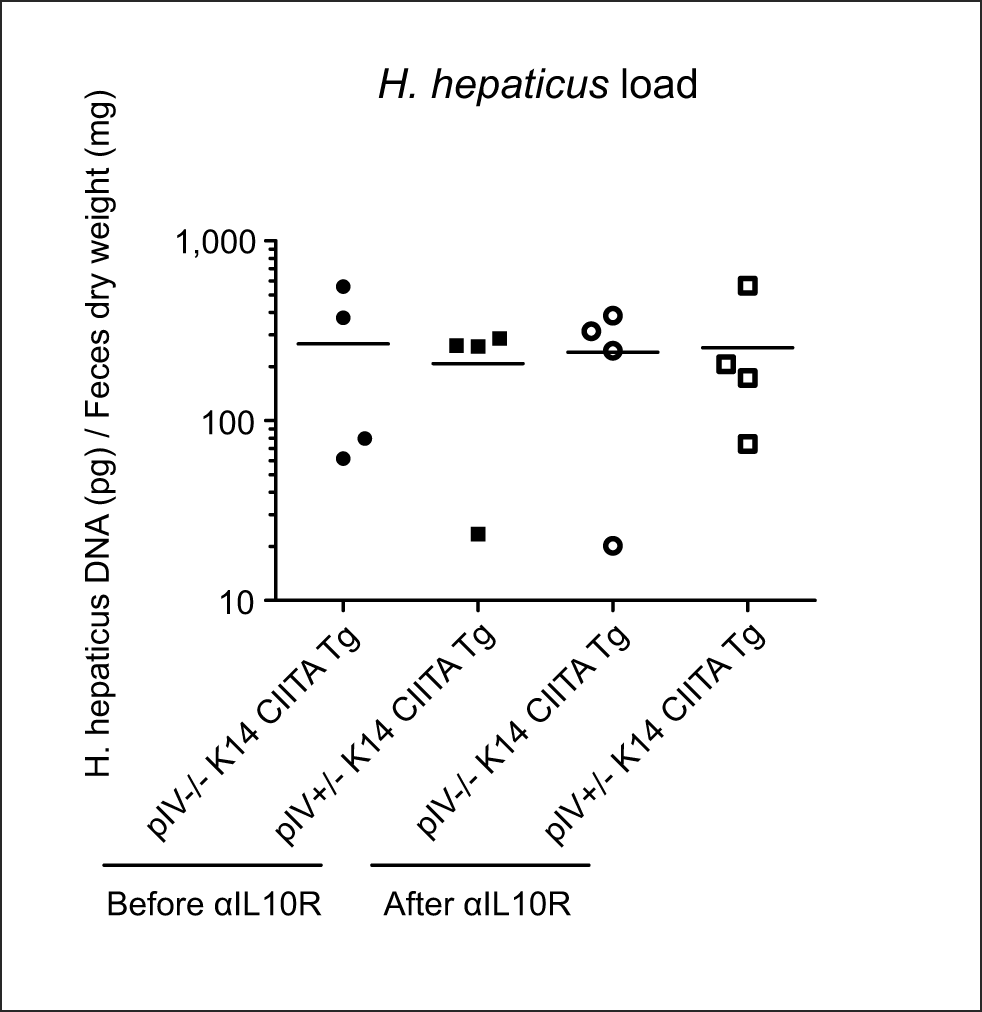

Supplement: Figure S4 — H. hepaticus colonization levels before and after anti-IL-10R treatment. Fresh fecal specimens from pIV−/− K14 CIITA Tg and pIV+/− K14 CIITA Tg mice were collected on days −4 to −2 before anti-IL-10R administration, and on days 26–28 of the experiment. Total fecal DNA was isolated and H. hepaticus DNA was quantified by qPCR and normalized to the dry weight of the fecal pellet. Each symbol represents a single animal. αIL10R, anti-interleukin-10 receptor monoclonal antibodies; (TIF) [file pone.0086844.s004.tif]

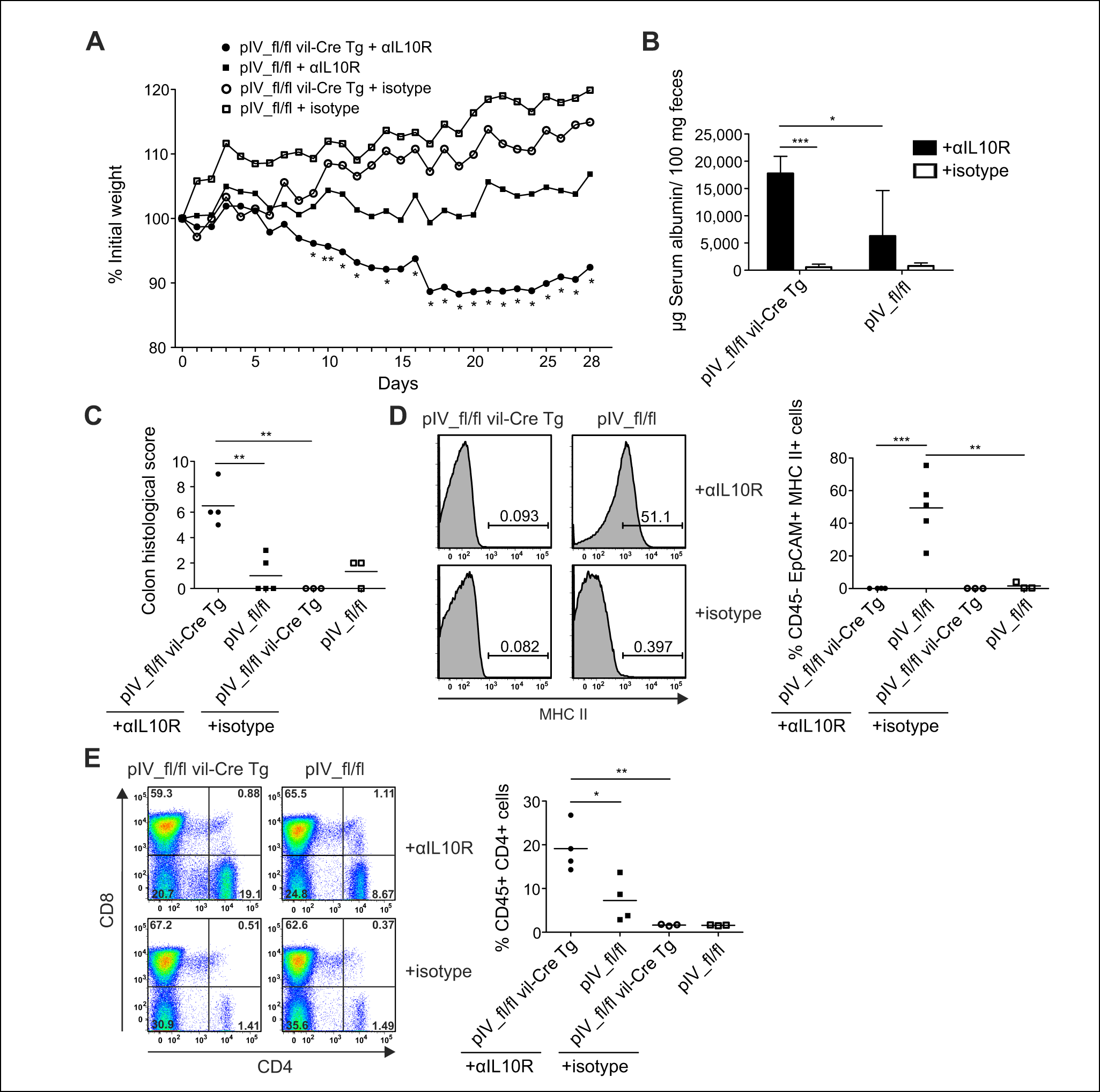

Supplement: Figure S5 — H. hepaticus infection plus anti-IL10R mAb treatment induces exacerbated colitis in pIVfl/fl vil-Cre Tg mice. (A) Development of body weight during anti-IL-10R mAb or isotype treatment of H. hepaticus-infected, tamoxifen-administered pIVfl/fl vil-Cre Tg mice or pIVfl/fl controls. Data are shown as mean. (B) Serum albumin concentrations in feces collected on days 26–30. Data are shown as mean and s.d. (C) Colitis scores upon organ collection on day 32. Data displayed as mean. (D) Frequency of CD45.2− EpCAM+ MHCII+ IECs. Representative histograms (left) and summarized data (right) as mean. (E) Frequency of CD3+ CD4+ and CD8+ T cells in the cIE. Representative histograms (left) and summarized data (right) as mean. All data represent n = 3–5 per group. αIL10R, anti-interleukin-10 receptor monoclonal antibodies; (TIF) [file pone.0086844.s005.tif]

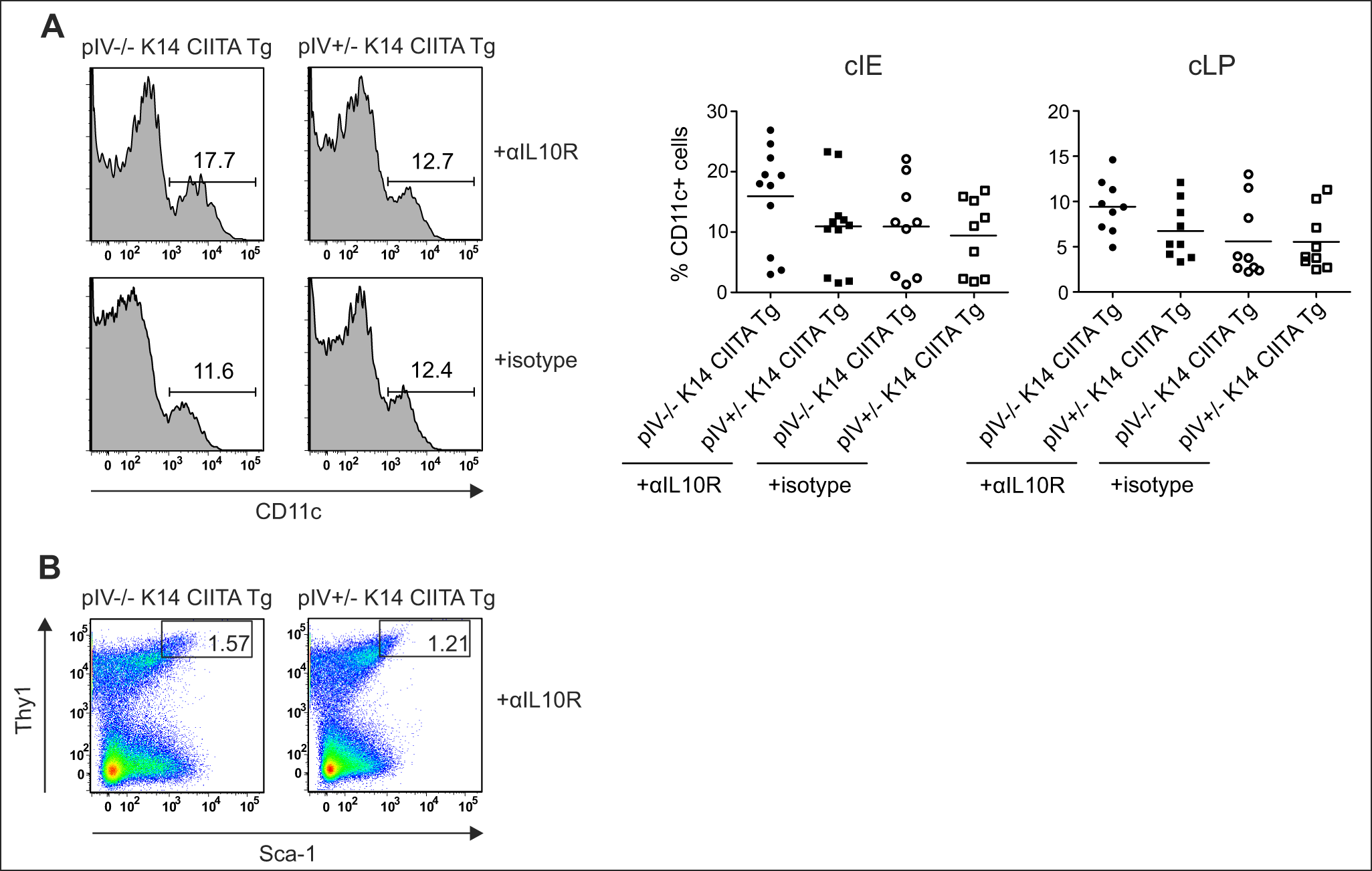

Supplement: Figure S6 — Levels of colonic DC and group 3 ILCs in colitic pIV−/− K14 CIITA Tg. (A) Frequency of Ly6C− CD11c+ conventional DCs in the cIE and cLP and (B) CD45.2+ Lin− (CD11b, Gr-1, B220) CD3ε− Thy1high Sca-1+ group 3 ILCs in the cLP isolated from anti-IL-10R mAb or isotype treated, H. hepaticus-infected pIV−/− K14 CIITA Tg mice or pIV+/− K14 CIITA Tg controls. (A) Representative histograms (left) display the cIE and data (right) represent mean (n = 9–11 per group) from three pooled experiments. (B) dot plots showed represent n = 3–4 per group. αIL10R, anti-interleukin-10 receptor monoclonal antibodies; (TIF) [file pone.0086844.s006.tif]

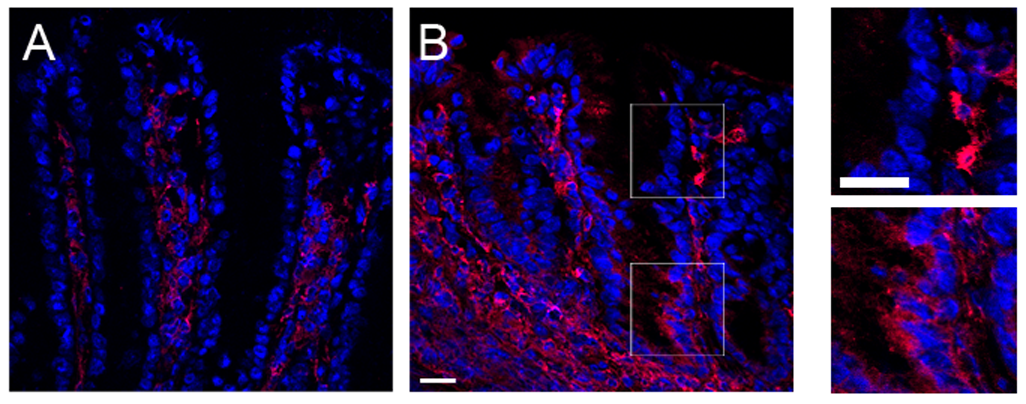

Supplement: Figure S7 — Colonic IECs from anti-IL-10R-treated pIV+/− K14 CIITA Tg mice express MHCII molecules apically and basolaterally. (A and B) H. hepaticus-infected pIV−/− K14 CIITA Tg or pIV+/− K14 CIITA Tg control mice were treated with anti-IL-10R mAb or isotype control mAb. Mid-colon sections were stained with DAPI (blue) to label nuclei and anti-MHCII mAb (red). (A) Representative pIV−/− K14 CIITA Tg mouse and (B) representative pIV+/− K14 CIITA Tg mouse. Right upper panel depicts a region with MHCII− IECs; Right lower panel depicts a region with MHCII+ IECs. Bar, 20 µm. (TIF) [file pone.0086844.s007.tif]

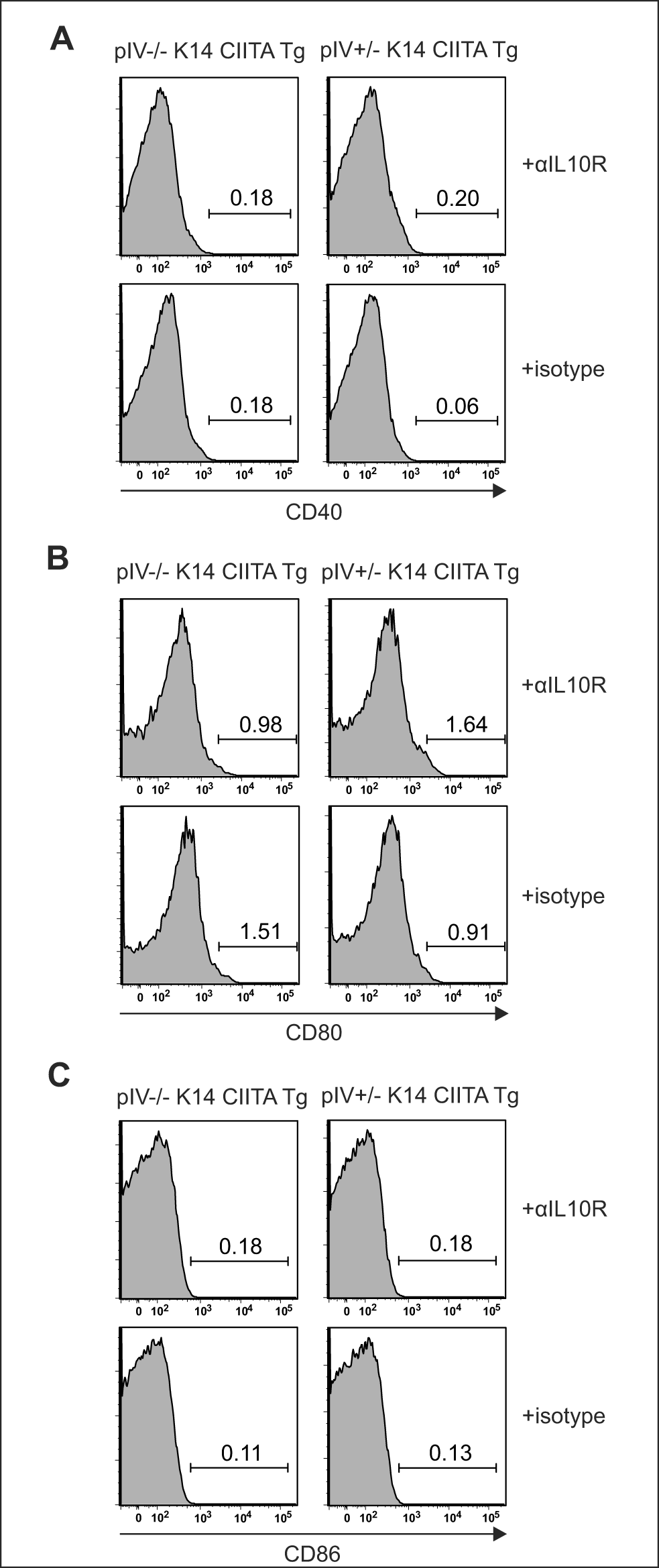

Supplement: Figure S8 — Colitic mice do not induce the expression of CD40, CD80 and CD86 on colonic IECs. (A–C) CD45.2− EpCAM+ IECs isolated from anti-IL-10R mAb or isotype treated, H. hepaticus-infected pIV−/− K14 CIITA Tg mice or pIV+/− K14 CIITA Tg controls were analysed for the expression of classical costimulatory molecules by flow cytometry. (A) Frequency of CD40, (B) CD80 or (C) CD86. Histograms represent n = 3–5 per group. αIL10R, anti-interleukin-10 receptor monoclonal antibodies; (TIF) [file pone.0086844.s008.tif]

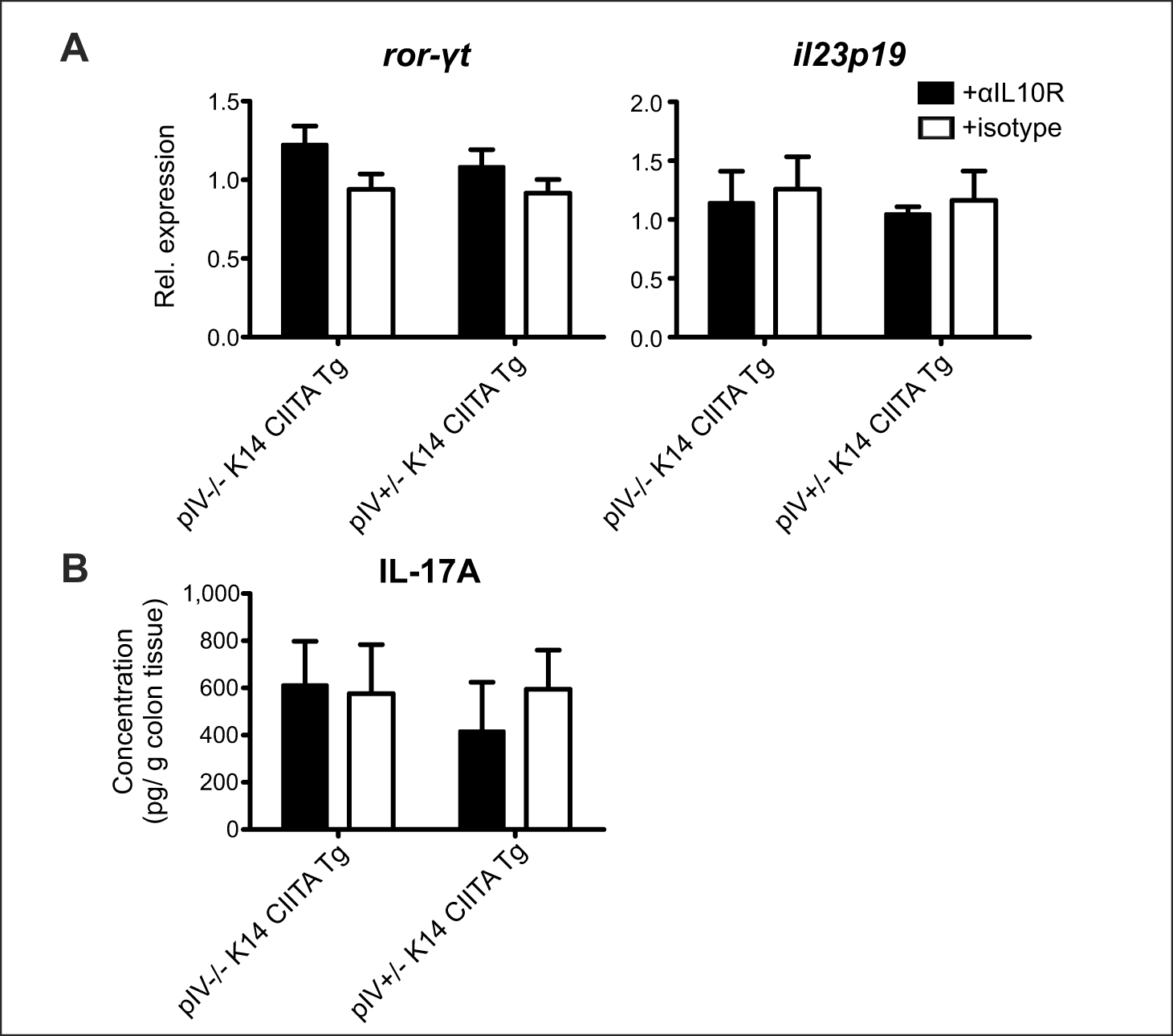

Supplement: Figure S9 — Expression levels of Th17- and group 3 ILC-associated factors. (A–B) H. hepaticus-infected pIV−/− K14 CIITA Tg or pIV+/− K14 CIITA Tg control mice were treated with anti-IL-10R mAb or isotype control mAb. (A) ror-γt and il23p19 mRNA expression levels in colon explants. Data represent n = 9–11 per group from three pooled experiments. (B) IL-17A secretion upon ex vivo organ culture of colon explants. Data represent n = 6 per group from two pooled experiments. Data displayed as mean and s.d. αIL10R, anti-interleukin-10 receptor monoclonal antibodies; (TIF) [file pone.0086844.s009.tif]
