# Supplementary material for: Interferon-γ Induces Expression of MHC Class II on Intestinal Epithelial Cells and Protects Mice from Colitis
Source: PLoS One. 2014 Jan 28;9(1):e86844. doi: 10.1371/journal.pone.0086844 (PMC3904943; doi:10.1371/journal.pone.0086844)
Supplement: Methods S1 — Additional methods applied to generate supporting figures. (DOC) [file pone.0086844.s010.doc]

**Methods S1**

**Immunisation model**

4-Hydroxy-3-nitrophenylacetyl hapten-conjugated chicken gamma globulin (NP_23_-CGG) or chicken ovalbumin was precipitated with 9 % aluminium potassium sulphate (alum) and resuspended in sterile PBS. Individual mice were immunised with 50 µg alum-precipitated protein s.c. on day 0 and boosted on day 35. A blood sample was collected weekly and purified serum was analysed by ELISA for the presence of specific IgG against either NP_4_ or ovalbumin as described previously [1].

**Quantification of fecal *Helicobacter hepaticus* load by qPCR**

Bacterial DNA was purified from fresh fecal specimen taken from mice chronically infected since birth with *H. hepaticus* using the DNA stool kit (Qiagen). *H. hepaticus* DNA was quantified by qPCR using primers specific for *H. hepaticus* 16S rRNA as described previously [2] and the SYBR Green Master Mix (Roche) on a LightCycler 480 machine (Roche). DNA Concentrations were obtained from a standard curve constructed using *H. hepaticus* DNA that was purified from bacterial cultures using the JETFLEX Genomic DNA Purification Kit (Genomed). Obtained concentrations were adjusted to the dry weight of fecal specimens.

**Tamoxifen preparation and administration**

Tamoxifen (Sigma-Aldrich) was dissolved in 100 % ethanol at 500 mg/ mL, and further diluted in pre-warmed sunflower seed oil (Sigma-Aldrich) at 10 mg/ mL. The solution was shaken for 3 h and vortexed every 30 min while being protected from light. To block MHCII expression specifically in IECs, pIV^fl/fl^ vil-Cre Tg and pIV^fl/fl^ littermates were injected i.p. with 1 mg tamoxifen/ 20 g mouse body weight on days -4, -3, -2, -1, 0, 17, 19 and 20 of the experiment.

**References for Methods S1**

1. Chevrier S, Genton C, Kallies A, Karnowski A, Otten LA, et al. (2009) CD93 is required for maintenance of antibody secretion and persistence of plasma cells in the bone marrow niche. Proc Natl Acad Sci U S A 106: 3895-3900.

2. Li X, Fox JG, Whary MT, Yan L, Shames B, et al. (1998) SCID/NCr mice naturally infected with Helicobacter hepaticus develop progressive hepatitis, proliferative typhlitis, and colitis. Infect Immun 66: 5477-5484.
